# Evaluation of sleep quality and daytime somnolence in patients with chronic obstructive pulmonary disease in pulmonary rehabilitation

**DOI:** 10.1186/s12890-020-1046-9

**Published:** 2020-01-15

**Authors:** Leandro Nobeschi, Juliana Zangirolami-Raimundo, Priscila Kessar Cordoni, Selma Denis Squassoni, Elie Fiss, Andrés Ricardo Pérez-Riera, Luiz Carlos de Abreu, Rodrigo Daminello Raimundo

**Affiliations:** Faculdade de Medicina do ABC, Av. Lauro Gomes, 2000 - Vila Sacadura Cabral, Santo André, São Paulo 09060-870 Brazil

**Keywords:** Chronic obstructive pulmonary disease, Quality of sleep, Pulmonary rehabilitation

## Abstract

**Background:**

Dyspnea, fatigue, and decline in sleep quality are symptoms of chronic obstructive pulmonary disease (COPD). Pulmonary rehabilitation programs have been shown to ameliorate dyspnea and fatigue. However, only a few studies have investigated the effects of pulmonary rehabilitation on the sleep quality of COPD patients. In this study, we analyzed the benefits of a pulmonary rehabilitation program to sleep quality and daytime somnolence in COPD patients.

**Methods:**

This study was a study of 30 moderate-severe COPD patients. All patients were evaluated by a pulmonologist and underwent polysomnography before participating in the study. For this study, we selected only ex-smokers and patients with sleep apnea were referred to the sleep clinic. These participants were prospectively recruited and not selected based on program completion. Before the start of the program, sleep quality and daytime somnolence of the participants were evaluated using the Pittsburgh Sleep Quality Index (PSQI) and the Epworth Sleepiness Scale (ESS), respectively. Rehabilitation program consisted of muscular training sessions conducted at the gym 3 times per week for 12 weeks. After rehabilitation program, the patients were reassessed and their sleep quality and daytime somnolence were reevaluated using the PSQI and the ESS, respectively.

**Results:**

Before rehabilitation, PSQI evaluation revealed that 73% of the participants had poor sleep quality, and ESS evaluation showed that 86.7% of the participants experienced daytime somnolence. After pulmonary rehabilitation, the PSQI specifically improved in terms of subjective sleep quality and sleep duration (< 0.001), habitual sleep efficiency (0.001), and sleep latency and sleep alterations (0.002) and there was also improvement in the ESS (< 0.001).

**Conclusion:**

Pulmonary rehabilitation program of gradually increasing intensity has the potential to provide sleep-related benefits to patients with COPD who have poor sleep quality and daytime somnolence.

**Trial registration:**

Registro Brasileiro de Ensaios Clínicos (ReBEC) RBR62b4z2.

## Background

Chronic obstructive pulmonary disease (COPD) is a disease characterized by limitation of airflow [1]. In terms of morbidity, it holds the fifth place in the world [2], and in terms of mortality, it is the third among non-communicable diseases [2, 3]. It is the main pneumopathy in Brazil [3]. COPD affects nearly 10% of adults aged 40 years and above [4], and the most significant risk factor of COPD is smoking [5, 6].

The disease is staged as follows: mild COPD when the forced expiratory volume in 1 s (FEV1) ≥80% of normal, moderate COPD when 50% ≤ FEV1 < 80% of normal, severe COPD when 30% ≤ FEV1 < 50% of normal, and very severe COPD when FEV1 < 30% of normal [7]. Low FEV1 is a risk factor for cardiovascular diseases. COPD causes limitation in ventilation, metabolism, and muscle capacity and is associated with reduction in muscle mass, exercise endurance, and strength of respiratory muscles [8]. The main complaints of COPD patients during consultations are dyspnea, fatigue, and reduction of sleep quality [9, 10]. Nearly 70% of COPD patients present with low sleep quality as determined using the Pittsburgh Sleep Quality Index (PSQI) evaluation [11]. Studies have shown that pulmonary rehabilitation programs improve muscle strength, respiratory patterns, and quality of life [12, 13]. However, only a few studies have reported results in sleep quality and daytime somnolence. Our study aimed to describe the benefits of a pulmonary rehabilitation program in terms of sleep quality and daytime somnolence in COPD patients.

## Methods

### Study site and ethical considerations

COPD patients were selected from the *Ambulatório de Reabilitação Pulmonar da Disciplina de Pneumologia da Faculdade de Medicina do ABC* (Outpatient Pulmonary Rehabilitation, Pneumology Discipline, ABC Medical School). All procedures related to the study were approved by the Ethics Committee for Research of the ABC Medical School (Certificate of Request for Ethical Evaluation: 57373016.3.0000.0082, report: 1627331). All participants provided signed informed consent. This study was recorded in Registro Brasileiro de Ensios Clínicos (ReBEC), trial registration: RBR62b4z2.

### Participants

These participants were prospectively recruited and not selected based on program completion. During the participant selection phase, 42 consecutively referred COPD patients were screened for eligibility with polysomnography and spirometry testing using a Koko PFT SpirometerTM (Spire, US). Each patient was given salbutamol (400 μg) to spirometry testing. Eligible patients had moderate to severe COPD (GOLD, 2014) [7] and a diagnosis of sleep apnea (> 15 OSA/hour). 30 patients comprised the final study population. BMI was calculated using the formula: weight (kg)/height2 (m^2^) and no patients were obese (> 30 kg/m^2^).

**Fig. 1:**
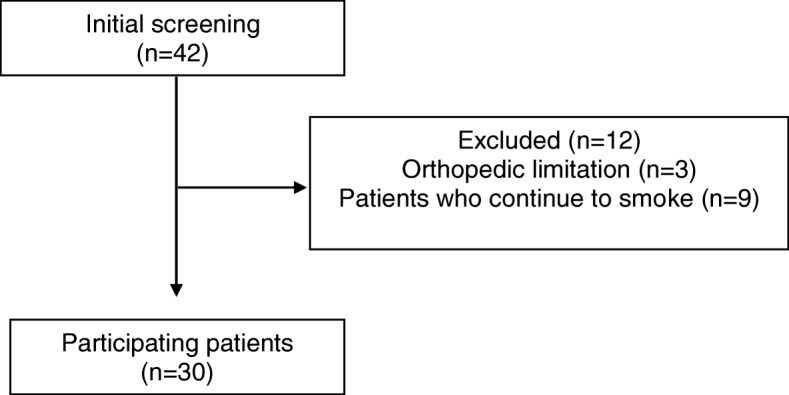
Flow chart of the study population

Exclusion criteria applied to the patient sample were as follows: patients who continue to smoke, inability to understand or perform the investigation procedures, orthopedic limitations that may interfere with participation in pulmonary rehabilitation program (Fig. 1).

### Evaluation of sleep quality and daytime somnolence

We used PSQI, validated for use in Portuguese, to evaluate sleep quality [14]. PSQI comprises 19 self-report questions categorized into 7 components and graded from 0 (no difficulty) to 3 (high difficulty). The components (C) of PSQI are subjective sleep quality (C1), sleep latency (C2), sleep duration (C3), habitual sleep efficiency (C4), sleep alterations (C5), use of medications to sleep (C6), and daytime sleep dysfunction (C7). The greater the sum of the values, the worse the sleep quality. We used a cutoff value of 5 or more in the final score (sum of components) to indicate reduction in sleep quality in accordance with the study by Buysse et al. [15] in which PSQI was developed. Question 8 of the PSQI evaluation (During the past month, how much of a problem has it been for you to keep up the enthusiasm to get things done?) was used to characterize the motivation of the participants.

We used Epworth Sleepiness Scale (ESS), validated for use in Portuguese and consisting of self-report questions, to evaluate daytime somnolence and verify how sleep may affect daytime activities. Excessive daytime sleepiness was defined as a condition characterized by an increased propensity to sleep in circumstances in which the affected individual and others would find inappropriate [16]. ESS assesses the likelihood of falling asleep in the following 8 daily situations: sitting and reading, sitting and watching television, inactively sitting in a public place (for example, a theater or a meeting), sitting in a car as a passenger for an hour without a break, lying down to rest in the afternoons when circumstances permit, sitting and talking to someone, sitting quietly after a lunch without alcohol, and sitting in a car stuck in traffic for a few minutes. Participant assigns a grade for each item ranging from 0 to 3 with the following meanings: grade 0 - participant would never doze off, grade 1 – participant has a slight chance of dozing off, grade 2 – participant has a moderate chance of dozing off, and grade 3 – participant has a high chance of dozing off. The overall score ranges from 0 to 24, with scores greater than 10 suggesting a diagnosis of excessive daytime sleepiness [16]. The patients completed the PSQI and ESS evaluations before and after undergoing the 12-week pulmonary rehabilitation program.

### Pulmonary rehabilitation protocol

Patients underwent 3 sessions of pulmonary rehabilitation per week for 12 consecutive weeks. The sessions were performed at the gym under the supervision of a physiotherapist or physical educator, who accompanied and encouraged the patients.

Rehabilitation program consisted of exercises for muscles of the upper and lower limbs. The selected exercises were performed at the *Estação de Johnson ST710* and were as follows: bench press, performed with a barbell 1.5 m in length, 1350 g in weight, with its own bench (Body and Soul, Brazil), and using weights ranging from 1 to 10 kg (according to the progress of the participant) to strengthen the pectoralis major muscle; lat pulldown, to strengthen the latissimus dorsi muscle; leg extension, to strengthen the quadriceps femoris muscle; and leg curls, to strengthen the hamstring muscles. The evaluators instructed all the patients before the start of the exercises on proper exercise technique, velocity, and width. Every patient warmed up without weights before properly performing the exercises.

One maximum repetition (1MR) was evaluated in every patient before the start of the training sessions. Intensities between 70 and 90% of 1MR may cause blood pressure increase [17]. Therefore, we decided that the exercises be performed at intensities of 60% of 1MR, which according to literature fulfills the goal of improving localized muscle endurance resistance [18]. In accordance with the study by Bernard et al., the exercises were performed in 3 sets of 8 to 10 repetitions, with 2-min intervals between sets [19]. Exercises were performed at maximum width and the exercise load was increased progressively during the sessions. Aerobic training was performed on a treadmill (T zero Johnson) and lasted for 30 min.

We monitored heart rate of the participants during the exercises using the Polar® RS800 CX heart monitor and a strip with electrodes placed on the chest, at the level of the xiphoid process. As a safety precaution, the pulmonary rehabilitation sessions were conducted at 70% of the maximum heart rate reached during the cardiopulmonary test.

### Statistical analysis

For statistical analysis, we use a paired T-test to compare PSQI and ESS before and after rehabilitation. Microsoft Excel 2013 was used to create the database, and the SPSS (Statistical Package for Social Research) software version 21.0 was used for statistical analysis. Shapiro-Wilk test was used to verify the normality of data. Descriptive statistics was presented in the form of the average and standard deviation. Data are reported as Median (minimum, maximum, 25th and 75th percentile) and compared between groups with Freidman’s test for repeated measures and with Wilcoxon’s test for paired measurements.

Linear regression was used to assess factors (weight, height, age, gender, and BMI) that influenced the PSQI in the pre-rehabilitation period. Spearman rank correlation was used to assess the relationship of spirometry parameters pre-rehabilitation PSQI and ESS scores.

## Results

All patients adhered to the pulmonary rehabilitation program for its whole duration of 12 weeks. Their characteristics are presented in Table 1: No patient was obese (BMI ≥ 30 kg/m2.) Table 2 shows the load progression in the exercises performed during the 12 weeks of pulmonary rehabilitation to strengthen the upper and lower limbs. There were progressive load increases between weeks 1 and 6 and from week 6 to week 12 with significant statistical differences (*p* <  0.001) observed in all the exercises.

**Table 1:** Characterization of the sample (*n* = 30)

Variables	Average	Standard deviation
Age (years)	68.17	6.11
Total body mass (kg)	67.50	13.58
Height (cm)	164.90	11.25
BMI (kg/m2)	24.85	4.71
Gender (%)		
Female	43.3	
Male	56.7	
Spirometry		
FEV1 (l)	1.32	0.54
FEV1(%)	49.90	17.70
FVC (l)	2.67	0.80
FVC (%)	78.70	19.50
FEV1/FVC	0.49	0.11
Cardiovascular		
Systolic BP (mmHg)	116.0	16.0
Diastolic BP (mmHg)	82.0	11.0
HR in rest (bpm)	92.5	13.1
Saturation in rest (%)	91.0	2.7
Smoking		
Never smoked (%)	5.5	
Former smoker (%)	94.5	
Co-morbidities		
Hypothyroidism (%)	3.3	
Heart failure (%)	16.6	
Ischemic heart disease (%)	10.0	
Hypertension (%)	26.6	
Arrhythmia (%)	0.0	
Depression (%)	6.7	
Diabetes (%)	13.3	
Time of COPD diagnosis		
1–5 years (%)	10.0	
6–10 years (%)	43.3	
≥ 11 years (%)	46.7	

*Kg* Kilograms, *m*^*2*^ square meters, *BMI* body mass index, *kg/m*^*2*^ kilograms per square meter, *BP* blood pressure, *mmHg* millimeters of mercury, *HR* heart rate, *bpm* beats per minute, *FEV*_*1*_ forced expiratory volume in the first second, *l* liter, *FVC* forced vital capacity

**Table 2:** Load evolution in exercises during pulmonary rehabilitation

	Week 1			Week 6			Week 12			*p*-value*
	Median	Minimum	Maximum	Median	Minimum	Maximum	Median	Minimum	Maximum	
Leg curl (Kg)	20.00	20	20	30.00	20	40	37.50	35	55	< 0.001
Bench press (Kg)	10.00	10	20	15.0	10	25	20.00	20	35	< 0.001
Lat pulldown (Kg)	10.00	6	10	12.00	8	14	14.00	12	18	< 0.001
Leg extension (Kg)	15.00	10	15	20.00	15	25	20.00	20	30	< 0.001

*Friedman test; *Kg* Kilograms

Sleep quality assessment using PSQI revealed poor sleep quality in 73% of the sample (final score average = 7.27) before rehabilitation and a 66% improvement in sleep quality (final score average = 4.83) after rehabilitation with a significant statistical difference (*p* <  0.001). The pre- and post-rehabilitation periods were compared in terms of subjective sleep quality, sleep latency, sleep duration, habitual sleep efficiency, and sleep alterations, and statistically significant differences were observed as shown in Table 3.

**Table 3:** Comparative Pittsburgh Sleep Quality Index (PSQI) and Epworth Sleepiness Scale (ESS) pre and pos rehabilitation

	Median	Percentis		Median	Percentiles		Median diff	*p*-value*
		25	75		25	75		
Subjective quality of sleep	1.00	1.00	1.00	1.00	0.00	1.00	0.00	< 0.001
Sleep latency	1.00	0.00	2.00	1.00	0.00	1.00	0.00	0.002
Sleep duration	1.00	0.00	3.00	1.00	0.00	2.00	0.00	< 0.001
Habitual sleep efficiency	1.00	1.00	2.00	1.00	0.00	1.00	0.00	0.001
Sleep alterations	1.00	1.00	2.00	1.00	1.00	1.00	0.00	0.002
Use of sleep medications	0.00	0.00	1.00	0.00	0.00	1.00	0.00	0.083
Daytime sleep dysfunction	0.00	0.00	1.00	0.00	0.00	1.00	0.00	0.083
Final score (PSQI)	6.5	4.75	8.75	4.00	3.00	6.25	2.50	< 0.001
Epworth scale (ESS)	14.00	11.00	16.00	10.5	7.00	13.25	3.50	< 0.001

*Wilcoxon test: before pulmonary rehabilitation (pre); after pulmonary rehabilitation (post); median difference pre minus post rehab (Median diff)

ESS showed that 86.7% of the sample presented pre-rehabilitation with daytime somnolence, which decreased to 53.3% post-rehabilitation with a statistically significant difference (*p* <  0.001) (Table 3).

The data suggests that patients who undergo pulmonary rehabilitation experience improvement in sleep quality and reduction in daytime somnolence.

There was no significant correlation between the pre-rehabilitation spirometry data and PSQI and ESS scores (Table 4).

**Table 4:** Spearman’s correlation coefficient (rho) of spirometry and Pittsburgh Sleep Quality Index (PSQI) and Epworth Sleepiness Scale (ESS) (ESS) in the pre-rehabilitation period

	FEV1 L/MIN	FVC L/MIN	FEV1FVC
FSa PSQI	−0.005	− 0.055	− 0.006
ESS	− 0.217	− 0.269	− 0.158

*FEV*_*1*_ Forced expiratory volume in the first second, *L* liter, *min* minute, *FVC* forced vital capacity, *FSa* final score average, *PSQI* Pittsburgh index, *ESS* Epworth scale

Before the start of the rehabilitation program, 28 patients (93%) answered “not during the past month,” and 2 patients (7%) answered “less than once a week” to question 8 (During the past month, how much trouble has it been for you to keep up the enthusiasm to get things done?) of PSQI questionnaire. At the end of rehabilitation, all the patients answered “not during the past month” to the same question.

Regression analysis shown in Table 5 reveals a significant finding of F(1.29) = 4.18 (*p* = 0.05) in the pre-rehabilitation period between the characteristic parameters of the sample and the PSQI. Regression analysis conducted in the post-rehabilitation period yielded similar results. Moreover, it showed an outcome prediction capacity of 9.9% (r^2^ = 0.099). The BMI variable remained significantly associated with the pre-rehabilitation final score of PSQI: β0–0.36 (*p* = 0.051).

**Table 5:** Linear regression analysis of PSQI with respect to patient characteristics

Variable	β (CI 95%)	p	Adjusted r^2^
Entry model			
Gender	0.161(− 2.416 4.544)	0.534	− 0.018
Weight	0.24(− 0.92 1.038)	0.902	
Height	−0.182(− 0.898 0.791)	0.896	
BMI	−0.639(−2.973 2.07)	0.715	
Age	0.046(−0.188 0.239)	0.809	
Final model			
BMI	−0.36(− 0.51 0.001)	0.051	0.099^a^

^a^ Linear regression analysis; *BMI* body mass index

## Discussion

This study aimed to analyze the benefits of a pulmonary rehabilitation program in terms of sleep quality and daytime somnolence in COPD patients. At the end of the pulmonary rehabilitation program, the patients experienced improvement in sleep quality (final score of PSQI < 0.001). Our study showed improvement specifically in subjective sleep quality and sleep duration (< 0.001), habitual sleep efficiency (0.001), sleep latency and sleep alterations (0.002), and use of sleep medication and daytime sleep dysfunction (0.083). We also found improvement in ESS (< 0.001). There was no correlation between spirometry and PSQI and ESS in the pre-rehabilitation period and no changes were found in the spirometry parameters in the post-rehabilitation period.

Our study revealed a correlation between BMI and sleep quality of participants; the higher the BMI, the worse the sleep quality, and the data correlated by reverse linear regression analysis.

We did not find a correlation between spirometry data and sleep quality. Patients with poor sleep quality also showed improvements in sleep quality, even when no changes were observed in spirometry. No participant in the sample missed any session of the pulmonary rehabilitation program. In analyzing the responses to question 8 of the PSQI questionnaire, we observed high levels of enthusiasm and motivation in the self-reports of the patients. During the pulmonary rehabilitation sessions, exercises for the upper and lower limbs were performed with progressively increasing loads, and a significant increase in the exercise load was observed over the 12 weeks of rehabilitation due to the assiduity of the participants. Our results showed statistically significant improvement in sleep quality, as seen in the PSQI components C1 to C5 (*p* < 0.005).

PSQI evaluation showed low sleep quality in COPD patients, which is consistent with reports in published literature [11, 20–24]. According to Soler et al., the poor sleep quality in COPD patients significantly affects their quality of life, which is still not explored in detail as seen in the guidelines of current pulmonary rehabilitation programs [22].

In our sample, 73% of the patients had low sleep quality (score ≥ 5 on PSQI evaluation) before undergoing the 12-week pulmonary rehabilitation program. After the program, 66% of the patients showed improvement in sleep quality (score ≤ 4 on PSQI evaluation), which differs from other studies that reported a reduction of PSQI score but not to the point of achieving an index of sleep quality improvement [22, 24].

Two important factors differentiate our study from earlier studies in published literature [22, 24]. The first factor is the duration of the rehabilitation program. The duration of the pulmonary rehabilitation programs in the above-mentioned studies was 8 weeks, while ours lasted to the end of the twelfth consecutive week. The 12-week duration of the treatment had a positive effect. A study involving COPD patients that participated in a pulmonary rehabilitation program with 2 sessions of lower limb strengthening exercises per week for 12 weeks using a cycle ergometer and respiratory training reported significant results in terms of sleep quality [25].

The second factor is the pulmonary rehabilitation program, which comprised training for upper and lower limbs on specific machines at the gym with standardized sets of maximum repetitions for each patient and enhanced the training intensity. We believe that the structure of the pulmonary rehabilitation program of gym exercises performed to maximum repetitions by each patient could potentially account for the discrepancy observed in the results. Studies show that the type, execution, and intensity of exercise may positively affect aerobic resistance and sleep quality, particularly sleep latency and sleep duration [26–29].

Pulmonary rehabilitation program we used in this study significantly enhanced muscle strength in patients by the sixth week of treatment. The high compliance rate of the participants who completed our pulmonary rehabilitation program is significant. Some studies reported problems with compliance and adherence to exercise routines in COPD patients [30]. In the pre-rehabilitation period, 86.7% of the patients in our sample experienced daytime somnolence, which is evaluated using the ESS. Studies have verified that, as the disease is chronic, COPD patients have a susceptibility to daytime somnolence [31]. Thus, the more severe the COPD, the greater the daytime somnolence [32].

Excessive daytime sleepiness occurs in 10% of the general population [33], and COPD patients typically present with the most common symptom of excessive daytime sleepiness [34]. A study on sleep apnea reported symptoms of fatigue, lack of energy, and drowsiness in 190 patients who participated in the study [35]. In COPD patients, fatigue is the second most common symptom, which considerably affects the quality of life, mostly in relation to dyspnea, depression, and insomnia [36]. In our study, we excluded patients with sleep apnea. Therefore, we consider the excessive daytime sleepiness experienced by patients in our sample to be related to poor sleep quality and dyspnea.

After the patients completed the pulmonary rehabilitation program, we observed improvement in the C7 component of PSQI (*p* = 0.083) and statistically significant improvement (*p* < 0.005) in daytime somnolence evaluated by ESS.

One limitation of our study is that we did not use a polysomnograph or actigraphy in the collection and analysis of data on sleep quality. The study findings are not generalizable to COPD patients with mild COPD and/or obstructive sleep apnoea, those who continue to smoke, and those who lack motivation and/or do not adhere to a complete pulmonary rehabilitation program, in addition, a selection bias may have occurred due to our type of patient recruitment. Another limitation is in relation to the mood changes, anxiety, and depression that are linked to poor sleep quality in COPD patients. The emotional factor was not considered a variable in our study, but according to published literature, physical exercise decreases anxiety and depression [37].

Larger studies are needed that include an external control group, use objective sleep measures such as actigraphy, and investigate for other modifiable factors such as depression/anxiety.

## Conclusion

We conclude that a pulmonary rehabilitation program of gradually increasing intensity has the potential to provide sleep-related benefits to patients with COPD who have poor sleep quality and daytime somnolence.

## Data Availability

The datasets analysed during the current study are available from the. corresponding author on reasonable request.

## Abbreviations

1MR: Maximum repetition
BMI: Body mass index
BP: Blood pressure
bpm: Beats per minute
COPD: Chronic obstructive pulmonary disease
ESS: Epworth Sleepiness Scale
FEV1: Forced expiratory volume in 1 s
FVC: Forced vital capacity
HR: Heart rate
Kg: Kilograms (Kg)
Kg/m^2^: Kilograms per square meter
l: Liter
m^2^: Square meters
mmHg: Millimeters of mercury
PSQI: Pittsburgh questionnaire index
SPSS: Statistical Package for Social Research
